# Exploring the Impact of TP53 Mutation and Wild-Type Status on the Efficacy of Immunotherapy in Non-Small Cell Lung Cancer

**DOI:** 10.3390/ijms26146939

**Published:** 2025-07-19

**Authors:** Alexander Yakobson, Ronen Brenner, Itamar Gothelf, Natalie Maimon Rabinovich, Ahron Yehonatan Cohen, Ashraf Abu Jama, Nashat Abu Yasin, Fahmi Abu Ghalion, Abed Agbarya, Walid Shalata

**Affiliations:** 1 The Legacy Heritage Cancer Center, Dr. Larry Norton Institute, Soroka Medical Center, Beer Sheva 84105, Israel; 2 Faculty of Health Sciences, Ben Gurion University of the Negev, Beer Sheva 84105, Israel; 3 Edith Wolfson Medical Center, Oncology Institute, Holon 58220, Israel; 4 Faculty of Medicine, Tel Aviv University, Tel Aviv 69978, Israel; 5 Goldman Medical School, Faculty of Health Sciences, Ben-Gurion University of the Negev, Beer Sheva 84105, Israel; 6 Department of Oncology, Meir Medical Center, Kfar Saba 44180, Israel; 7 Oncology Department, Bnai Zion Medical Center, Haifa 31048, Israel

**Keywords:** lung cancer, immunotherapy, tumor protein P53 (TP53), NSCLC, real-world evidence

## Abstract

TP (tumor protein) 53 mutation status plays a critical role in cancer progression and may influence survival outcomes in non-small cell lung cancer (NSCLC) patients receiving immunotherapy. This study investigates the impact of TP53 mutation status and immunotherapy treatment on survival in NSCLC patients. This retrospective study analyzed NSCLC patients treated with pembrolizumab or ipilimumab plus nivolumab, stratified by TP53 mutation status and PD-L1 (programmed death-ligand 1) expression (<1%, 1–49%, >50%). Survival outcomes (overall survival (OS) and progression free survival (PFS) were assessed using Kaplan–Meier curves and log-rank tests, with subgroup analysis by histological subtype. In squamous cell cancer (SCC) patients, no significant differences in OS or PFS were found based on TP53 mutation status or treatment type. A trend toward improved survival was observed with pembrolizumab (*p* = 0.088). In adenocarcinoma patients, significant differences in OS and PFS were observed based on TP53 mutation status. Pembrolizumab showed superior survival outcomes compared to ipilimumab plus nivolumab in TP53 wild-type patients (*p* < 0.001). PD-L1 ≥ 1% also predicted better outcomes, especially in adenocarcinoma patients. TP53 mutation status and immunotherapy type significantly influence survival outcomes in NSCLC, particularly in adenocarcinoma patients. Pembrolizumab demonstrated superior efficacy in TP53 wild-type patients, with PD-L1 expression further refining survival predictions. These findings underscore the importance of personalized treatment strategies based on TP53 status and PD-L1 expression in NSCLC. Further studies are needed to validate these results and optimize treatment approaches.

## 1. Introduction

Lung cancer remains one of the most prevalent and deadly cancers worldwide, posing a significant public health challenge. It is the leading cause of cancer-related deaths, with an estimated 2.2 million new cases and 1.8 million deaths annually. The majority of lung cancer patients, over two-thirds, are diagnosed at age 65 or older, with only a small minority (3%) being under 40. This age demographic, coupled with the fact that most cases are diagnosed when the cancer has already metastasized beyond the lungs, significantly contributes to the high morbidity and mortality rates associated with the illness [1,2,3,4,5].

Histologically, lung cancer is broadly categorized into small cell lung cancer (SCLC), accounting for 15–20% of cases, and non-small cell lung cancer (NSCLC), which represents the vast majority at 80–85%. NSCLC itself is further subtyped, with adenocarcinoma being the most common (45–50% of cases), followed by squamous cell carcinoma (30–35%), large-cell carcinoma (10–15%), and other less frequent types (around 5%). The poor prognosis of NSCLC, in particular, stems from the alarming statistic that approximately 70% of patients are diagnosed at advanced stages (III–IV), leading to a median survival period of only about one year [2,3,4,5,6,7].

The incidence of lung cancer varies globally, with higher rates in countries with higher smoking prevalence, although the disease is increasingly diagnosed in non-smokers as well. Risk factors such as tobacco use, occupational exposures, and genetic predisposition contribute to the development of lung cancer, but environmental factors and air pollution are also gaining attention as emerging causes. The geographical variations in lung cancer incidence and mortality highlight the global burden of the disease. China, the United States, and Japan report the highest numbers of new cases and deaths. When considering age-standardized rates, Hungary, New Caledonia, China, Serbia, and French Polynesia show the highest incidence per 100,000 people [5,6,7,8].

Immune checkpoint inhibitors (ICIs) are widely used as first-line treatment for non-oncogene-addicted NSCLC. Recent trials have demonstrated that the value of programmed death ligand-1 (PD-L1) serves as a predictive factor for survival when ICIs are used whether as monotherapy or in combination therapy of chemo-immunotherapy compared to chemotherapy alone in patients with advanced NSCLC [8,9,10,11,12].

Many genes are implicated in the onset and progression of NSCLC, with tumor protein P53 (TP53) being one of the most well-known and widely studied. Reports have shown that the occurrence of TP53 mutations in primary tumors and metastatic lymph nodes in NSCLC was 23.2% and 21.4%, respectively, with reported frequencies varying by geographical region [12,13,14,15,16].

One study reported an overall prevalence of 45.2% in NSCLC, with 25.0% in adenocarcinomas and 63.6% in squamous cell carcinomas. Another study documented TP53 mutation rates ranging from 40% to 46% specifically in adenocarcinomas [17,18,19].

TP53 is located on chromosome 17p and encodes the p53 protein. When TP53 is mutated, p53 loses its ability to regulate cell growth, apoptosis, and DNA repair processes, contributing to the initiation and progression of tumors. Approximately 80% of TP53 mutations are missense mutations, while the remaining mutations include frameshifts, truncations, and deletions [13,14,15,16,17,18].

TP53, a gene frequently mutated in NSCLC, contains 11 exons, with exons 2 to 11 encoding a 393-amino acid protein known as p53. Under normal conditions, various stress signals can activate the p53 signaling pathway, triggering multiple transcriptional programs. Cell cycle arrest is primarily mediated by p21, a checkpoint protein that inhibits cyclin-dependent kinases. DNA repair is facilitated by genes such as POLH, RRM2B, and PCNA, while apoptosis is triggered through both intrinsic and extrinsic signaling pathways. Additionally, p53 regulates numerous other cellular processes. Inactivation of TP53 leads to increased cell proliferation, survival, invasion, and contributes to cancer progression [19,20,21,22].

The prognostic impact of TP53 mutations in patients treated with ICIs is not well understood, and no studies have assessed the outcomes of TP53 mutations in patients receiving a combination of platinum-based chemotherapy and an ICI (such as pembrolizumab, or ipilimumab plus nivolumab). With the recent approval of these combinations, there is significant variability in first-line treatment approaches. Therefore, our study aims to investigate the prognostic impact of TP53 mutations in patients undergoing standard first-line treatment for advanced metastatic NSCLC.

## 2. Results

A total of 346 patients participated in this study (Table 1). Patients had a median age of 67 years, with a range from 37 to 87 years. The majority of the patients were male (68.5%), and most had adenocarcinoma (67.3%) as the histology. Smoking history showed that 50.4% were current smokers, 35.9% were past smokers, and 13% were never smokers. The ECOG performance status distribution was 20.9% in ECOG 0, 58% in ECOG 1, and 21.2% in ECOG 2+. Regarding immunotherapy, 59.2% received pembrolizumab, and 40.8% received ipilimumab plus nivolumab. PD-L1 expression in the overall population was distributed as 36.7% with <1%, 26.0% with 1–49%, and 37.3% with >50%.

The TP53 mutation group consists of 79 participants, representing 22.83% of the total group. The median age of participants in this group is 67.4 years, with ages ranging from 41 to 86 years. In terms of gender, 27.8% are female and 72.2% are male. Histologically, 65.8% of participants have adenocarcinoma, 31.6% have squamous cell carcinoma, and 2.6% have other types. No participants in this group have adenosquamous carcinoma. Regarding smoking status, 6.3% of participants are never smokers, A total of 49.3% are current smokers, 43.0% are past smokers, and 1.4% have unknown smoking status. In terms of ECOG performance status, 24% have an ECOG score of 0, 52% have a score of 1, and 24% have a score of 2 or higher. For immunotherapy, 65.8% of participants received pembrolizumab, while 7.8% received ipilimumab plus nivolumab. Regarding PD-L1 expression, 34.1% have PD-L1 expression of less than 1%, 19% have expression between 1% and 49%, and 46.9% have expression greater than 50%.

In contrast, the 267 patients with TP53 wild-type (77.17%) had a median age of 66.9 years, with a range from 37 to 87 years. Among this group, 32.7% were female, and 67.3% were male. Histologically, most patients had adenocarcinoma (68.3%), followed by squamous cell carcinoma (26.4%). The smoking status showed that 39.04% were current smokers and 26.01% were past smokers. For ECOG, 59.6% were in ECOG 1, 19.8% in ECOG 0, and 20.6% in ECOG 2+. Immunotherapy was administered to 57.4% of patients with pembrolizumab and 42.6% with ipilimumab plus nivolumab. Regarding PD-L1 expression, 37.5% had <1%, 34.5% had 1–49%, and 28% had ≥50%.

**Figure 1:**
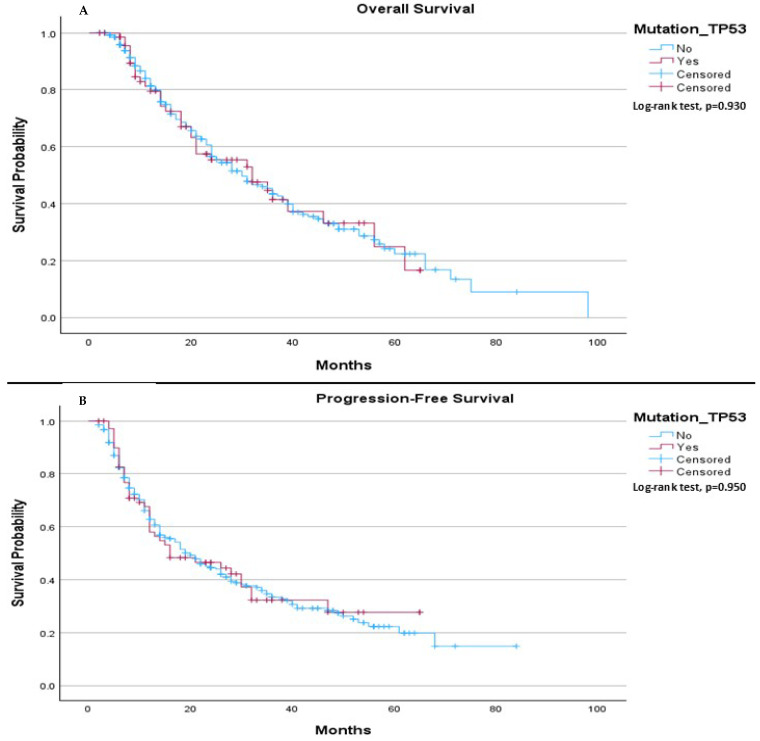
Comparison of median OS and PFS between patients with and without TP53 mutation. (**A**) The median OS was 30.0 months (95% CI: 23.73 to 36.27) for patients without the TP53 mutation and 32.0 months (95% CI: 17.86 to 46.15) for patients with the TP53 mutation. The log-rank test indicated no significant survival difference between the groups (*p* = 0.930). (**B**) The median progression-free survival was 20.0 months (95% CI: 15.40 to 24.60) for patients without the TP53 mutation and 16.0 months (95% CI: 2.73 to 29.27) for patients with the TP53 mutation. The log-rank test showed no significant difference between the two groups (*p* = 0.950).

The median OS in our cohort was 30.0 months for patients without the TP53 mutation and 32.0 months for those with the TP53 mutation, (*p* = 0.930), (Figure 1A). The median PFS was 20.0 months for patients without the TP53 mutation, compared to 16.0 months for patients with the mutation, (*p* = 0.950), (Figure 1B).

**Figure 2:**
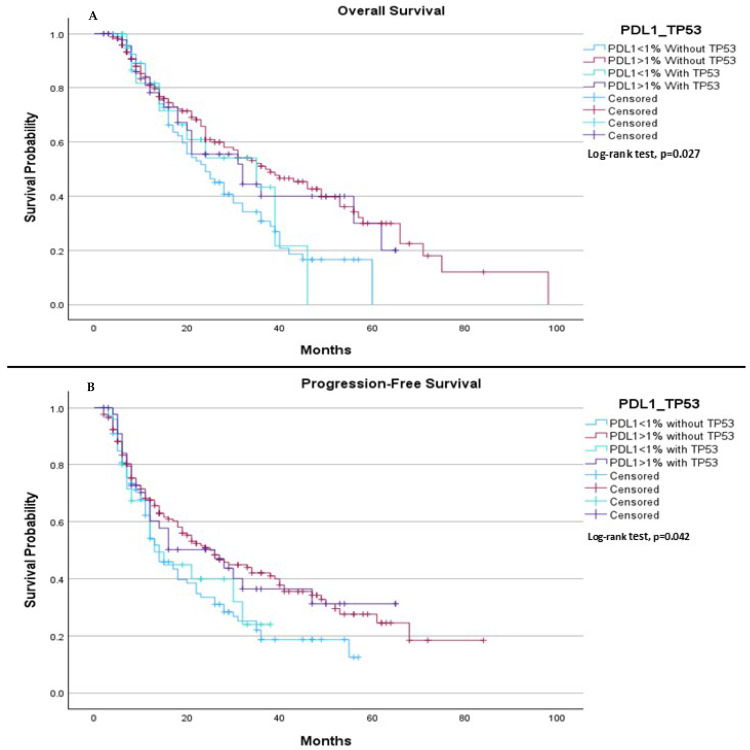
Comparison of median OS and PFS based on PD-L1 expression and TP53 mutation status. (**A**) The median OS was 24.0 months (95% CI: 18.08 to 29.92) for patients with PD-L1 < 1% without TP53 mutation, 37.0 months (95% CI: 27.06 to 46.95) for patients with PD-L1 ≥ 1% without TP53 mutation, 35.0 months (95% CI: 12.56 to 57.44) for patients with PD-L1 < 1% with TP53 mutation, and 32.0 months (95% CI: 15.11 to 48.89) for patients with PD-L1 ≥ 1% with TP53 mutation. The log-rank test indicated a statistically significant survival difference between the groups (*p* = 0.027). (**B**) Progression-Free Survival: The median progression-free survival was 14.0 months (95% CI: 10.71 to 17.29) for patients with PD-L1 < 1% without TP53 mutation, 26.0 months (95% CI: 19.03 to 32.97) for patients with PD-L1 ≥ 1% without TP53 mutation, 13.0 months (95% CI: 6.66 to 19.34) for patients with PD-L1 < 1% with TP53 mutation, and 26.0 months (95% CI: 10.56 to 41.44) for patients with PD-L1 ≥ 1% with TP53 mutation. The log-rank test showed a statistically significant difference between the groups (*p* = 0.042).

When comparing all groups based on PD-L1 expression (<1% or ≥1%) and TP53 mutation status (mutation or wild type), we observed a significant difference in both median OS and median PFS. The median OS was 24.0 months for patients with PD-L1 < 1% without the TP53 mutation, 37.0 months for those with PD-L1 ≥ 1% without the TP53 mutation, 35.0 months for patients with PD-L1 < 1% with the TP53 mutation, and 32.0 months for patients with PD-L1 ≥ 1% with the TP53 mutation, (*p* = 0.027), (Figure 2A). Regarding PFS, the median was 14.0 months for patients with PD-L1 < 1% without the TP53 mutation, 26.0 months for those with PD-L1 ≥ 1% without the TP53 mutation, 13.0 months for patients with PD-L1 < 1% with the TP53 mutation, and 26.0 months for those with PD-L1 ≥ 1% with the TP53 mutation, (*p* = 0.042), (Figure 2B).

**Figure 3:**
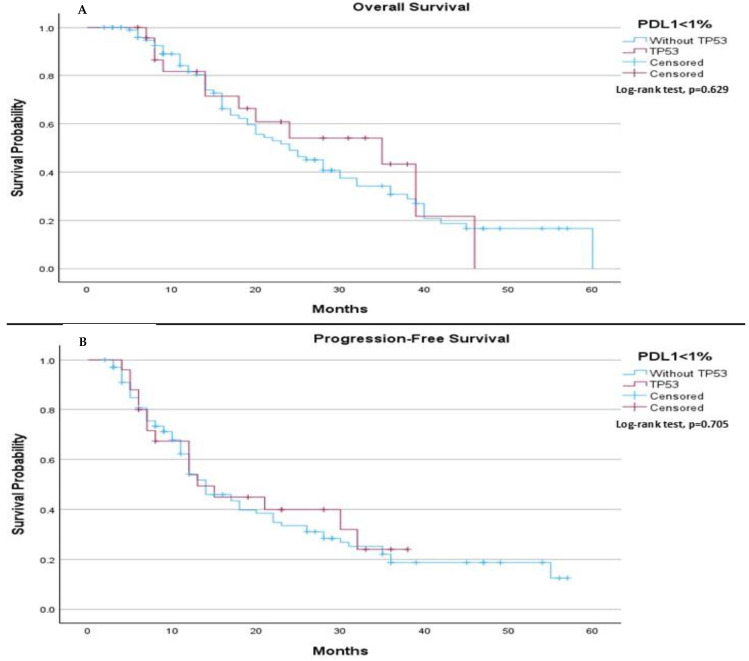
Subgroup analysis of median OS and PFS in PD-L1 negative (>1%) patients based on TP53 mutation status. (**A**) The median OS was 24.0 months (95% CI: 18.08 to 29.92) for patients without the TP53 mutation and 35.0 months (95% CI: 12.56 to 57.44) for patients with the TP53 mutation. The log-rank test indicated no significant survival difference between the groups (*p* = 0.629). (**B**) The median PFS was 14.0 months (95% CI: 10.71 to 17.29) for patients without the TP53 mutation and 13.0 months (95% CI: 6.66 to 19.34) for patients with the TP53 mutation. The log-rank test showed no significant difference between the two groups (*p* = 0.705).

In subgroups’ analysis regarding to PDL-1 expressions (<1%, 1–49%, and >50%), in PDL-1 negative patients, no significant differences in median OS or median PFS were observed between those with and without the TP53 mutation. The median OS was 24.0 months (95% CI: 18.08 to 29.92) for patients without the TP53 mutation and 35.0 months (95% CI: 12.56 to 57.44) for those with the mutation (*p*-value 0.629), (Figure 3A). Similarly, the median PFS was 14.0 months for patients without the TP53 mutation and 13.0 months for those with the mutation (*p*-value 0.705), (Figure 3B).

**Figure 4:**
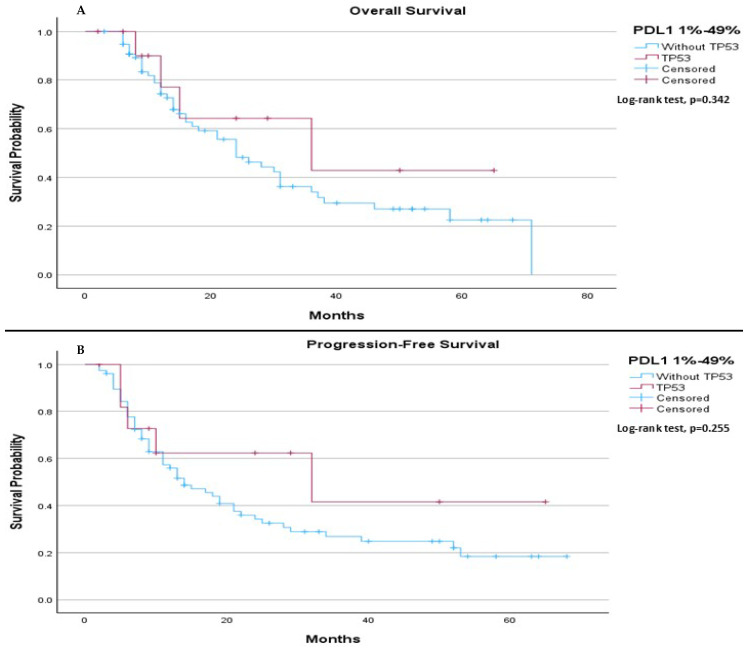
Subgroup analysis of median OS and PFS in patients with PD-L1 expression between 1–49% based on TP53 mutation status. (**A**) The median OS was 24.0 months (95% CI: 16.22 to 31.78) for patients without the TP53 mutation and 36.0 months (95% CI: 0.00 to 75.90) for patients with the TP53 mutation. The log-rank test indicated no significant survival difference between the groups (*p* = 0.342). (**B**) The median PFS was 14.0 months (95% CI: 8.24 to 19.76) for patients without the TP53 mutation and 32.0 months (95% CI: 0.00 to 72.87) for patients with the TP53 mutation. The log-rank test showed no significant difference between the two groups (*p* = 0.255).

In patients with PDL-1 expression between 1–49%, there was no significant difference in median OS or median PFS between those with and without the TP53 mutation. The median OS was 24.0 months for patients without the TP53 mutation and 36.0 months for those with the mutation (*p*-value 0.342), (Figure 4A). Similarly, the median PFS was 14.0 months (95% CI: 8.24 to 19.76) for patients without the TP53 mutation and 32.0 months for those with the mutation (*p*-value 0.255), (Figure 4B).

**Figure 5:**
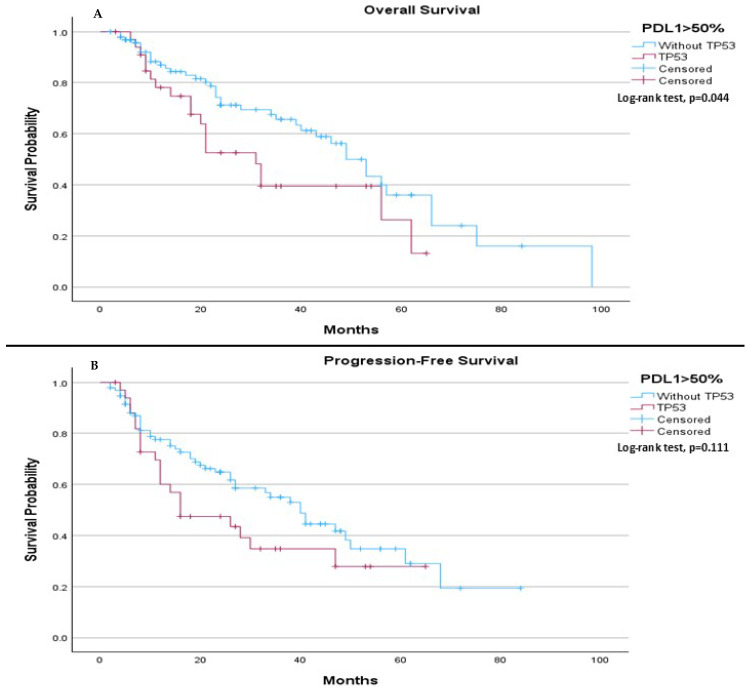
Subgroup analysis of median OS and PFS in patients with PD-L1 expression > 50% based on TP53 mutation status. (**A**) The median OS was 49.0 months (95% CI 41.354 to 56.646) for patients without TP53 mutation and 31.0 months (95% CI 21.803 to 40.197) for those with TP53 mutation. The log-rank test showed a significant difference in survival between the two groups (*p* = 0.044). (**B**) The median PFS was 40.0 months (95% CI 31.967 to 48.033) for patients without TP53 mutation and 16.0 months (95% CI 0.570 to 31.430) for those with TP53 mutation. The log-rank test showed no statistically significant difference between the two groups (*p* = 0.111).

In patients with PDL-1 expression > 50%, TP53 mutation status was associated with a significant difference in median OS, with those without the mutation having a longer median OS (49.0 months) compared to those with the mutation (31.0 months, *p* = 0.044), (Figure 5A). However, no significant difference was observed in median PFS, with a median of 40.0 months for patients without the TP53 mutation and 16.0 months for those with the mutation (*p* = 0.111), (Figure 5B).

**Figure 6:**
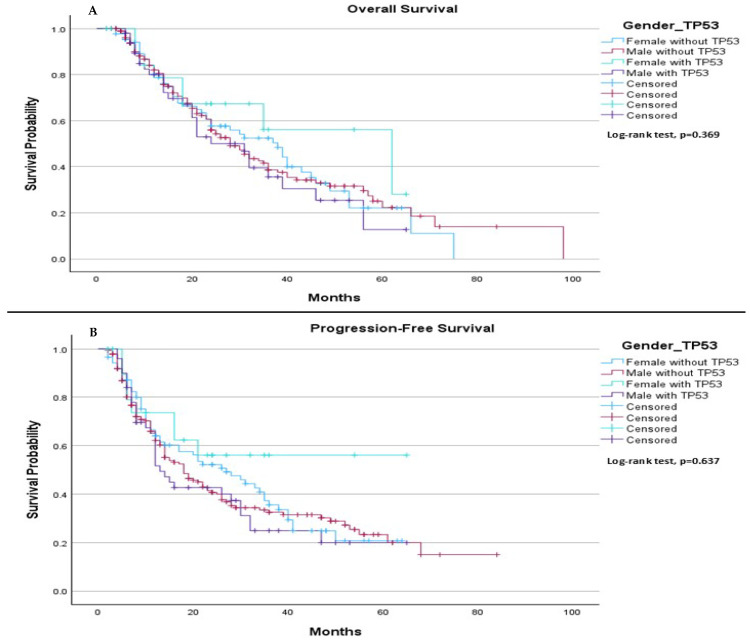
Comparison of median OS and PFS based on gender and TP53 mutation status. (**A**) The median OS was 38.0 months (95% CI: 26.69 to 49.31) for females without TP53, 28.0 months (95% CI: 22.53 to 33.47) for males without TP53, 62.0 months (95% CI: 22.40 to 101.60) for females with TP53, and 31.0 months (95% CI: 22.15 to 39.85) for males with TP53. The log-rank test indicated no statistically significant difference in survival distributions between the groups (*p* = 0.369). (**B**) The median PFS was 27.0 months (95% CI: 16.78 to 37.22) for females without TP53, 18.0 months (95% CI: 12.99 to 23.01) for males without TP53, while median survival was not estimable for females with TP53. For males with TP53, the median progression-free survival was 13.0 months (95% CI: 10.16 to 15.84). The log-rank test showed no statistically significant difference between the groups (*p* = 0.637).

When comparing survival outcomes based on gender, we found no significant difference in either median OS or median PFS between individuals with and without the TP53 mutation. Specifically, the median OS was 38.0 months for females without TP53, 28.0 months for males without TP53, 62.0 months for females with TP53, and 31.0 months for males with TP53 (*p* = 0.369), (Figure 6A). Regarding median PFS, it was 27.0 months for females without TP53 and 18.0 months for males without TP53. For females with TP53, the median PFS was not estimable, while for males with TP53, the median PFS was 13.0 months (*p* = 0.637), (Figure 6B).

**Figure 7:**
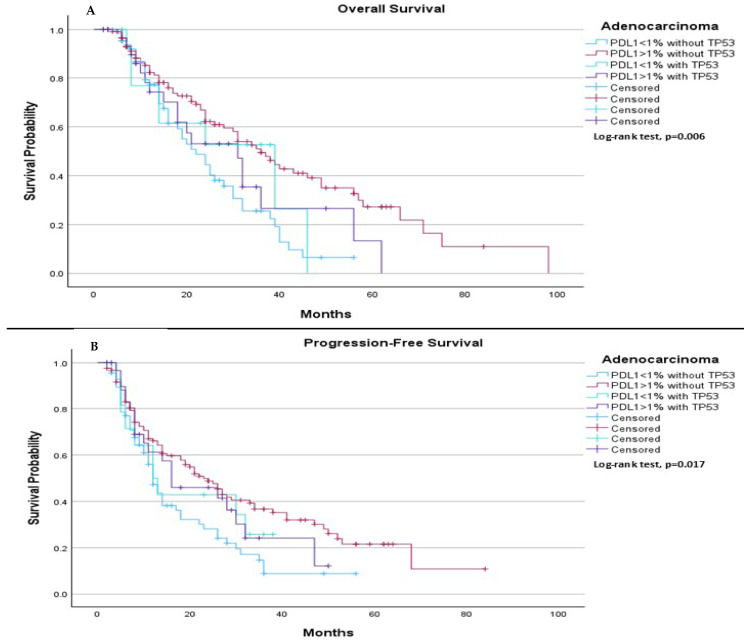
Comparison of median OS and PFS based on histological subtype, PDL-1 expression levels, and TP53 mutation status in adenocarcinoma patients. (**A**) Among adenocarcinoma patients, the median OS was 22.0 months (95% CI: 15.49 to 28.52) for those with PDL-1 < 1% without TP53, 36.0 months (95% CI: 28.90 to 43.10) for those with PDL1 ≥ 1% without TP53, 39.0 months (95% CI: 11.21 to 66.79) for those with PDL1 < 1% with TP53, and 31.0 months (95% CI: 19.81 to 42.19) for those with PDL1 ≥ 1% with TP53. The log-rank test indicated a statistically significant survival difference between the groups (*p* = 0.006). (**B**) Among adenocarcinoma patients, the median PFS was 12.0 months (95% CI: 9.98 to 14.02) for those with PDL1 < 1% without TP53, 23.0 months (95% CI: 17.15 to 28.85) for those with PDL1 ≥ 1% without TP53, 12.0 months (95% CI: 5.89 to 18.11) for those with PDL1 < 1% with TP53, and 16.0 months (95% CI: 2.02 to 29.98) for those with PDL1 ≥ 1% with TP53. The log-rank test showed a statistically significant difference between the groups (*p* = 0.017).

When comparing survival outcomes based on subtype of histology, among adenocarcinoma patients, we observed significant differences in both median PFS and median OS depending on both PDL-1 expression and TP53 mutation status. The median OS was 22.0 months for patients with PDL-1 < 1% without TP53, 36.0 months for those with PDL-1 ≥ 1% without TP53, 39.0 months for those with PDL-1 < 1% with TP53, and 31.0 months for those with PDL-1 ≥ 1% with TP53 (*p* = 0.006), (Figure 7A). Regarding median PFS, it was 12.0 months for those with PDL-1 < 1% without TP53, 23.0 months for those with PDL-1 ≥ 1% without TP53, 12.0 months for those with PDL-1 < 1% with TP53, and 16.0 months for those with PDL-1 ≥ 1% with TP53 (*p* = 0.017), (Figure 7B).

**Figure 8:**
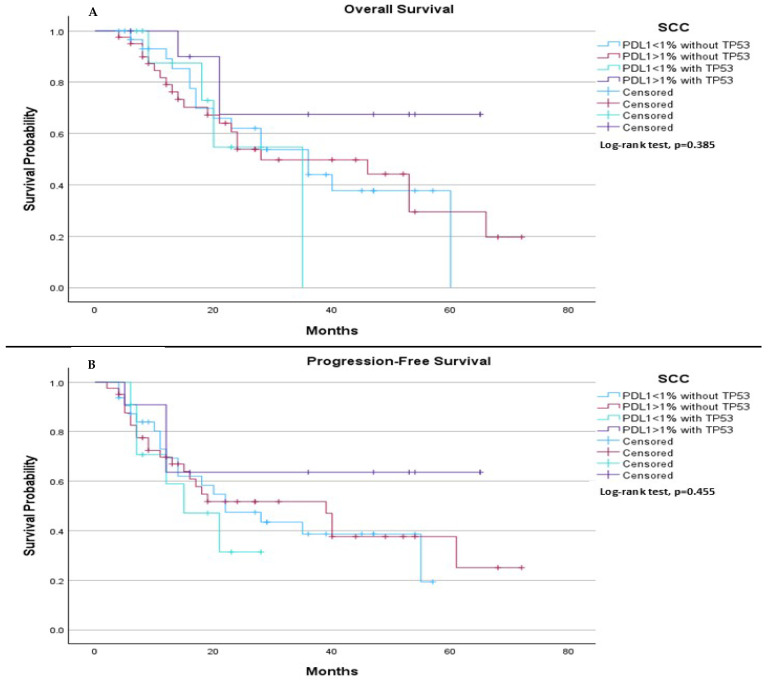
Comparison of median OS and PFS based on PDL-1 expression levels and TP53 mutation status in SCC patients. (**A**) Among SCC patients, the median overall survival was 36.0 months (95% CI: 21.59 to 50.42) for those with PDL1 < 1% without TP53, 28.0 months (95% CI: 3.45 to 52.55) for those with PDL1 ≥ 1% without TP53, 35.0 months (95% CI: not estimable) for those with PDL1 < 1% with TP53, and not estimable for those with PDL1 ≥ 1% with TP53. The log-rank test indicated no statistically significant survival difference between the groups (*p* = 0.385). (**B**) Among SCC patients, the median PFS was 22.0 months (95% CI: 9.58 to 34.42) for those with PDL1 < 1% without TP53, 39.0 months (95% CI: 19.32 to 58.67) for those with PDL1 ≥ 1% without TP53, 15.0 months (95% CI: 4.34 to 25.66) for those with PDL1 < 1% with TP53, and not estimable for those with PDL1 ≥ 1% with TP53. The log-rank test showed no statistically significant difference between the groups (*p* = 0.455).

On the other hand, among SCC patients, we observed no significant differences in both median PFS and median OS. Specifically, the median OS was 36.0 months for patients with PDL-1 < 1% without TP53, 28.0 months for those with PDL-1 ≥ 1% without TP53, 35.0 months for those with PDL-1 < 1% with TP53, and was not estimable for those with PDL-1 ≥ 1% with TP53 (*p* = 0.385), (Figure 8A). Regarding median PFS, it was 22.0 months for those with PDL-1 < 1% without TP53, 39.0 months for those with PDL-1 ≥ 1% without TP53, 15.0 months for those with PDL-1 < 1% with TP53, and was not estimable for those with PDL-1 ≥ 1% with TP53 (*p* = 0.455), (Figure 8B).

**Figure 9:**
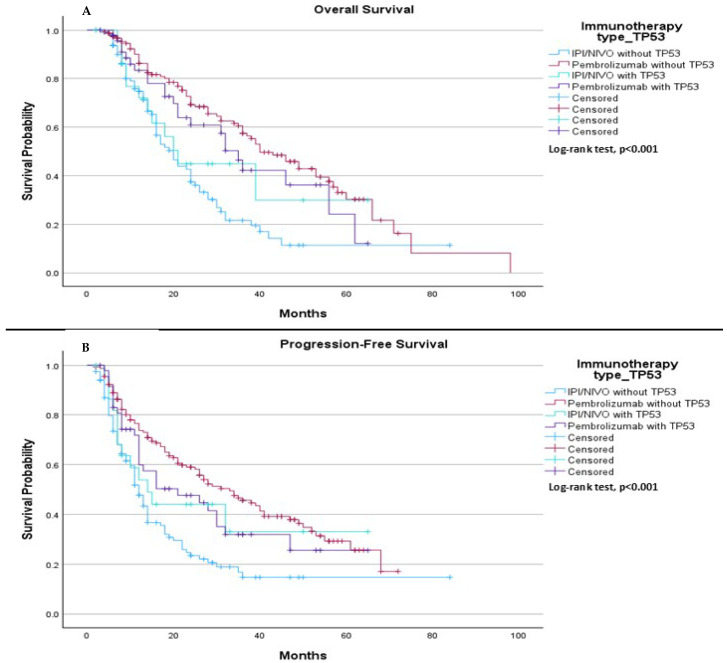
Comparison of median OS and PFS based on TP53 mutation status and immunotherapy treatment (pembrolizumab vs. ipilimumab plus nivolumab (IPI/NIVO)). (**A**) The median OS was 20.0 months (95% CI: 15.87 to 24.14) for patients treated with ipilimumab plus nivolumab without TP53 mutation, 40.0 months (95% CI: 31.60 to 48.40) for patients treated with pembrolizumab without TP53 mutation, 21.0 months (95% CI: 15.04 to 26.96) for patients treated with ipilimumab plus nivolumab with TP53 mutation, and 35.0 months (95% CI: 29.35 to 40.65) for patients treated with pembrolizumab with TP53 mutation. The log-rank test indicated a statistically significant survival difference between the groups (*p* < 0.001). (**B**) The median PFS was 12.0 months (95% CI: 10.24 to 13.75) for patients treated with ipilimumab plus nivolumab without TP53 mutation, 33.0 months (95% CI: 23.78 to 42.22) for patients treated with pembrolizumab without TP53 mutation, 14.0 months (95% CI: 6.76 to 21.23) for patients treated with ipilimumab plus nivolumab with TP53 mutation, and 21.0 months (95% CI: 5.79 to 36.21) for patients treated with pembrolizumab with TP53 mutation. The log-rank test showed a statistically significant difference between the groups (*p* < 0.001).

When comparing TP53 mutation status (mutation or wild type) with the type of immunotherapy treatment (pembrolizumab vs. ipilimumab plus nivolumab), we observed a significant difference in median OS. The median OS was 20.0 months for patients treated with ipilimumab plus nivolumab without the TP53 mutation, 40.0 months for those treated with pembrolizumab without the TP53 mutation, 21.0 months for patients treated with ipilimumab plus nivolumab with the TP53 mutation, and 35.0 months for patients treated with Pembrolizumab with the TP53 mutation, (*p* < 0.001), (Figure 9A). In terms of PFS, the median was 12.0 months for patients treated with ipilimumab plus nivolumab without the TP53 mutation, 33.0 months for those treated with pembrolizumab without the TP53 mutation, 14.0 months for patients treated with ipilimumab plus nivolumab with the TP53 mutation, and 21.0 months for those treated with pembrolizumab with the TP53 mutation, (*p* < 0.001), (Figure 9B).

**Figure 10:**
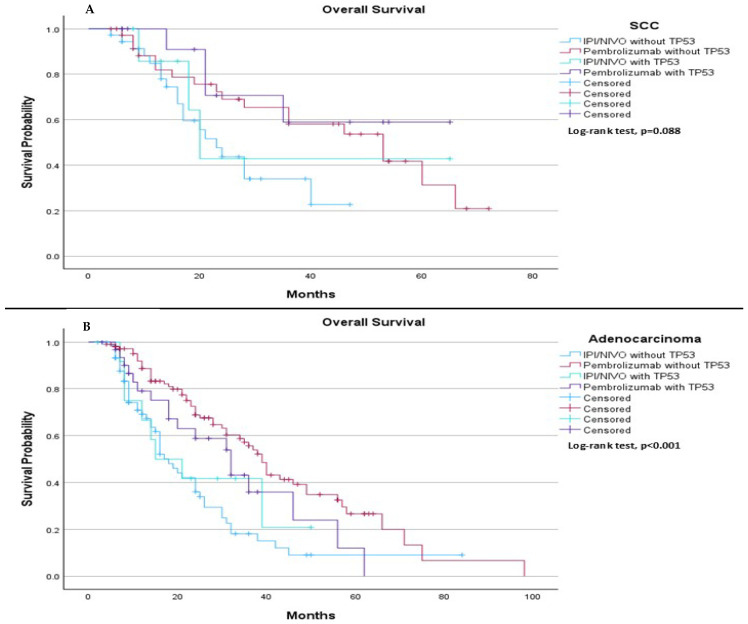
Comparison of median overall survival (OS) based on TP53 mutation status and immunotherapy treatment (pembrolizumab vs. ipilimumab plus nivolumab (IPI/NIVO)) in adenocarcinoma and SCC patients. (**A**) Among patients with squamous cell carcinoma, the median overall survival was 23.0 months (95% CI: 16.70 to 29.30) for those treated with ipilimumab plus nivolumab without TP53 mutation, 53.0 months (95% CI: 31.87 to 74.12) for those treated with pembrolizumab without TP53 mutation, 20.0 months (95% CI: 15.90 to 24.10) for those treated with ipilimumab plus nivolumab with TP53 mutation, and not estimable for those treated with pembrolizumab with TP53 mutation. The log-rank test indicated no statistically significant but it suggested a trend in survival difference between the groups (*p* = 0.088). (**B**) Among adenocarcinoma patients, the median OS was 18.0 months (95% CI: 14.33 to 21.67) for those treated with ipilimumab plus nivolumab without TP53 mutation, 39.0 months (95% CI: 35.28 to 42.73) for those treated with pembrolizumab without TP53 mutation, 15.0 months (95% CI: 3.12 to 26.88) for those treated with ipilimumab plus nivolumab with TP53 mutation, and 32.0 months (95% CI: 21.47 to 42.53) for those treated with pembrolizumab with TP53 mutation. The log-rank test indicated a statistically significant survival difference between the groups (*p* < 0.001).

When comparing TP53 mutation status (mutation vs. wild type) with the type of immunotherapy treatment (pembrolizumab vs. ipilimumab plus nivolumab), we observed distinct trends and significant differences based on histological subtype. Among SCC patients, the results suggested a trend in median OS, with a median OS of 23.0 months for those treated with IPI/NIVO without TP53 mutation, 53.0 months for those treated with pembrolizumab without TP53 mutation, 20.0 months for those treated with ipilimumab plus nivolumab with TP53 mutation, and not estimable for those treated with pembrolizumab with TP53 mutation (*p* = 0.088), (Figure 10A). In contrast, among adenocarcinoma patients, significant differences were observed in median OS, with a median OS of 18.0 months for those treated with ipilimumab plus nivolumab without TP53 mutation, 39.0 months for those treated with pembrolizumab without TP53 mutation, 15.0 months for those treated with ipilimumab plus nivolumab with TP53 mutation, and 32.0 months for those treated with pembrolizumab with TP53 mutation (*p* < 0.001), (Figure 10B).

## 3. Discussion

In the field of precision and personalized cancer therapy, precise tumor classification—encompassing both histological subtypes and molecular pathological characteristics—is critical.

PD-L1 is an immune checkpoint protein that inhibits T-cell activity by binding to its receptor, PD-1. ICIs, including PD-1 and PD-L1 inhibitors, work by blocking this interaction, thereby reactivating T-cell–mediated immune responses against tumor cells. High PD-L1 expression may suggest that tumor cells are more likely to respond to ICIs. Since PD-L1 detection methods are well established, PD-L1 has become a practical and accessible clinical biomarker. PD-L1 expression can be quantified using various scoring methods, including tumor proportion score (TPS), tumor cells (TC), immune cells (IC), and combined positive score (CPS). TPS refers to the percentage of viable tumor cells showing partial or complete membrane staining for PD-L1 out of all viable tumor cells in the sample. In contrast, CPS incorporates PD-L1 expression on both tumor cells and tumor-associated immune cells within the tumor microenvironment, offering a broader assessment of PD-L1 activity [23,24,25,26].

The influence of TP53 mutation status on survival outcomes for NSCLC patients receiving chemo-immunotherapy remains an important area of investigation, though it is not yet fully understood in both clinical trials and real-world settings. To our knowledge, this is the first study to evaluate the impact of TP53 mutation in first-line, locally advanced, and metastatic squamous cell carcinoma and non-squamous NSCLC patients treated with a combination of chemo-immunotherapy. Our research focused on the prognostic effect of TP53 mutation status on OS and PFS in a real-world cohort of patients with advanced or metastatic adenocarcinoma and squamous cell carcinoma. Univariable analysis revealed no significant improvement in either median PFS or OS between TP53 mutation and wild-type groups.

Multivariable analysis confirmed these findings. In subgroup analysis, including variables such as histological subtype (adenocarcinoma and SCC), treatment type (pembrolizumab and ipilimumab plus nivolumab), and TP53 mutation status, patients with PD-L1 expression > 50% showed significant differences in both OS and PFS when treated with chemo-immunotherapy. On the other hand, no differences were observed in OS or PFS based on gender (male vs. female) or PD-L1 expression levels of 1–49% and <1%.

When comparing our results to the Check-Mate 9LA [27], KEYNOTE-189 [28], and KEYNOTE-407 [29] trials, we observed some notable trends regarding TP53 status (mutation vs. wild type). In our study, the comparison of immunotherapy regimens for NSCLC patients revealed distinct patterns in median OS and median PFS based on both TP53 mutation status and the type of immunotherapy used. For patients with SCC, there was a trend toward OS benefit for the pembrolizumab group (*p* = 0.088); however, pembrolizumab showed a trend toward better OS for those without TP53 mutation. In contrast, for patients with adenocarcinoma, pembrolizumab provided a statistically significant improvement in OS compared to ipilimumab plus nivolumab, regardless of TP53 mutation status (*p* < 0.001). Regarding PFS, pembrolizumab also demonstrated a significant advantage over ipilimumab plus nivolumab in both the SCC and adenocarcinoma subgroups (*p* < 0.001). This trend was consistent across both TP53 wild-type and mutant cohorts. Based on the findings, pembrolizumab appears to be the superior immunotherapy treatment for patients with adenocarcinoma, particularly in those without TP53 mutation, showing longer median OS and median PFS. In comparing our results to those of the main clinical trials, the findings from the CheckMate 9LA trial showed no statistically significant differences in median OS for either adenocarcinoma or SCC, irrespective of TP53 status. Similarly, the KEYNOTE-189 and KEYNOTE-407 trials also did not demonstrate significant improvements in OS based on TP53 status [27,28,29]. However, in a pooled analysis from the German National Network Genomic Medicine Lung Cancer, which evaluated first-line treatment outcomes with pembrolizumab in non-squamous PD-L1 ≥ 50% patients according to KRAS/TP53 mutational status, it was noted that G12C/TP53-mutated patients experienced the longest PFS and OS (33.7 and 65.3 months, respectively; *p* = 0.002). The similarity between our cohort and the German cohort lies in the fact that both were derived from similar geographical and environmental settings, which may contribute to the observed outcomes [14]. While clinical trials typically include patients from diverse regions, the shared local environment, and potentially similar genetic backgrounds in both cohorts may offer a more consistent basis for comparison. This could help explain the comparable results observed in our study and the German study, suggesting that regional and environmental factors may play a role in influencing patient responses to treatment, regardless of the differences in clinical trial settings.

A meta-analysis of first-line treatment options for PD-L1–negative NSCLC concluded that the combination of nivolumab, ipilimumab, and chemotherapy was likely the most effective treatment based on treatment rankings [27]. In addition, real-world data indicated that for metastatic NSCLC patients with PD-L1 expression < 1%, the combination of nivolumab, ipilimumab, and chemotherapy showed numerically better treatment responses compared to pembrolizumab plus chemotherapy [30,31]. Conversely, other real-world data highlighted that in the low PD-L1 group, the median OS was 16 months for patients treated with ipilimumab and nivolumab, compared to 12 months for those treated with pembrolizumab (*p* = 0.02) [11]. Despite these findings, it remains unclear which treatment approach—chemotherapy combined with either mono-immunotherapy (pembrolizumab) or chemo-immunotherapy (ipilimumab plus nivolumab)—is the optimal first-line therapy. This uncertainty may be why many physicians prefer treating patients with TP53 mutations or PD-L1 < 1% expression with ipilimumab plus nivolumab, as it is thought to address the more aggressive mutation [30,31]. However, our findings suggest that TP53 mutation status does not significantly affect survival outcomes in PD-L1 negative patients or those with PD-L1 expression of 1–49%, nor does it appear to influence median PFS. While TP53 mutation may impact median OS in high PD-L1 expressing patients, it does not seem to have an effect on PFS. Furthermore, in patients with PD-L1 expression > 1% or <1% and different TP53 mutation statuses, our results indicate that both PD-L1 expression and TP53 mutation status influence median OS and PFS outcomes in this cohort.

It is well established in the literature that TP53 mutations in lung cancer do not significantly differ in frequency between males and females [32,33,34]. However, in our study, we observed a higher prevalence of TP53 mutations among male patients. This discrepancy may be attributed to geographical and environmental factors, particularly smoking behavior. In our cohort, the majority of male patients were smokers, whereas smoking is relatively rare among women in our region. Given the known association between smoking and increased risk of TP53 mutations, this may explain the higher mutation rate observed in males in our study. Additionally, it is noteworthy that in our cohort, patients with positive PD-L1 expression—known to be more prevalent among smokers—were also more likely to harbor TP53 mutations, consistent with findings reported in the literature [35].

This study has several limitations. First, being a retrospective analysis, it is susceptible to selection bias and incomplete data. Second, the data were collected from only three institutions, which may limit the generalizability of the findings to a broader population. Additionally, the relatively small sample size in certain subgroups may reduce the statistical power of some analyses. To improve the robustness and generalizability of these findings, future research should expand its scope by incorporating data from multiple centers or even different countries, as well as including a larger cohort of patients. Moreover, since this study compares only two types of immune checkpoint inhibitors (pembrolizumab vs. nivolumab plus ipilimumab), future investigations should consider examining a wider range of ICIs and their effects. Conducting multi-center, international studies with more extensive patient cohorts will be crucial to validating and refining these results.

## 4. Materials and Methods

### 4.1. Cohort Selection (Study Population)

This retrospective, non-interventional observational study was conducted across multiple institutions, focusing on patients diagnosed with NSCLC and TP53 mutations who received chemo-immunotherapy (whether as pembrolizumab or ipilimumab plus nivolumab) as their first-line treatment between January 2018 and January 2024. Data collection continued until September 2024, the date of the last follow-up. The data gathered included patient demographics (age at diagnosis, sex), treatment regimen, OS, PFS, therapy start and end dates, smoking history, metastasis sites, PD-L1 values, date of last follow-up, progression sites; and Eastern Cooperative Oncology Group (ECOG) performance status scores.

All patients were evaluated by a multidisciplinary team, which included radiologists, medical oncologists, radiation oncologists, pulmonologists, pathologists, thoracic surgeons, and nuclear medicine physicians. These evaluations were based on pathological findings, imaging results, and the patient’s performance status. A primary physician was assigned to each patient, responsible for overseeing their treatment. For patients diagnosed with advanced or metastatic disease (any T, N 1–3, and/or M1), medical oncologists took the lead in managing their care, following the treatment guidelines established by the National Comprehensive Cancer Network (NCCN) [22], in addition the reporting of this study conforms to the observational studies statement.

### 4.2. Treatment and Protocols Administrated

#### 4.2.1. Patients Diagnosed with Adenocarcinoma

Each treatment cycle, lasting three weeks, began with intravenous administration of cisplatin at 75 mg per square meter of body-surface area or carboplatin, with the dosage adjusted according to the area under the concentration-time curve (AUC of 6–4), based on the patient’s performance status. This was paired with pemetrexed, dosed at 500 mg per square meter of body-surface area. In addition, nivolumab was given intravenously at 360 mg every three weeks, and ipilimumab was administered at 1 mg/kg every six weeks. After the first two cycles, the platinum-based chemotherapy agent was discontinued, and pemetrexed, nivolumab, and ipilimumab continued as maintenance therapy, in accordance with the Checkmate 9LA trial protocol [27]. Alternatively, pembrolizumab was used instead of nivolumab and ipilimumab, after the initial four cycles, with the platinum agent discontinued, following the Keynote 189 [28] trial regimen. Pembrolizumab was administered intravenously at 200 mg every three weeks, alongside pemetrexed. The treatment was planned to continue for up to two years or until the development of unacceptable toxicity or disease progression. All patients were premedicated with folic acid, vitamin B12, and glucocorticoids in line with institutional guidelines before receiving pemetrexed.

#### 4.2.2. Squamous Cell Carcinoma

Each treatment cycle, lasting three weeks, began with intravenous administration of cisplatin at 75 mg per square meter of body-surface area or carboplatin, adjusted based on the AUC of 4–6, depending on the patient’s performance status. This was combined with paclitaxel at 175 mg per square meter of body-surface area. Additionally, nivolumab was administered intravenously at a dose of 360 mg every three weeks, and ipilimumab at 1 mg/kg every six weeks. Following the first two cycles, chemotherapy agents (paclitaxel and platinum) were discontinued, and nivolumab and ipilimumab continued as maintenance immunotherapy, following the regimen outlined in the Checkmate 9LA trial [27]. Alternatively, pembrolizumab was used in place of nivolumab and ipilimumab after the initial four cycles, with the chemotherapy agents discontinued. Pembrolizumab was administered intravenously at a dose of 200 mg every three weeks, as per the Keynote 407 trial protocol [29]. This treatment regimen was intended to be administered for up to two years or until disease progression or unacceptable toxicity occurred.

### 4.3. Inclusion Criteria

Patients were eligible for inclusion in the study if they met the following criteria: Age 18 years or older and had a histologically confirmed diagnosis of NSCLC without driver mutations.

### 4.4. Exclusion Criteria

Patients were excluded from the study if they had received any systemic cancer therapy within the four years preceding the study. This exclusion was necessary to prevent previous treatments from affecting the study’s results.

### 4.5. Patient Monitoring, Clinical Information, and Data Collection

Before starting treatment, patients underwent disease staging, which included brain magnetic resonance imaging (MRI), total-body computed tomography (CT) scans, or fluorodeoxyglucose (FDG) positron emission tomography-computed tomography (PET-CT). Measurable target lesions, including those with NSCLC epidermal growth factor receptor (EGFR) mutations identified through follow-up or imaging, were evaluated. Oncologists assessed treatment responses using the Response Evaluation Criteria in Solid Tumors (RECIST). Safety monitoring was performed according to the Common Terminology Criteria for Adverse Events (CTCAE), version 5.0. Throughout the treatment period, radiologic assessments, including brain MRI, CT, or FDG-PET-CT, were conducted every three to four months.

### 4.6. Statistical Analysis

Descriptive statistics were used to summarize the baseline demographic, clinical, and molecular characteristics of the patients. Continuous data with non-normal distributions were presented as medians (range), while categorical variables were reported as frequencies (percentages). Kaplan–Meier survival analyses were conducted to evaluate OS and PFS based on the presence or absence of TP53 mutations. These analyses were further stratified by key clinical factors, including PD-L1 expression levels, type of ICI, and histological diagnosis. Log-rank tests were performed to assess the statistical significance of differences in survival distributions. Multivariable analyses for PFS and OS were carried out using Cox proportional hazard regression models to estimate hazard ratios (HRs) with their corresponding 95% confidence intervals (CIs). All *p*-values were two-sided, with a significance threshold set at *p* < 0.05. The statistical analyses were conducted using SPSS software, version 29.0.

## 5. Conclusions

This study underscores the potential impact of TP53 mutation status and the type of immunotherapy treatment on survival outcomes in both SCC and adenocarcinoma lung cancer patients. Our findings suggest that both PD-L1 expression (whether >1% or <1%) and TP53 mutation status play a crucial role in influencing median PFS and OS outcomes in this cohort. The log-rank test revealed a statistically significant difference in survival between the subgroups. Specifically, for adenocarcinoma patients, our results indicate that PD-L1 expression levels and TP53 mutation status are significant factors affecting both OS and PFS, highlighting their potential relevance in predicting patient prognosis.

## Figures and Tables

**Table 1:** Baseline characteristics of the study cohort.

Characteristics	Overall (%), N = 346	TP53 Mutation (%), N = 79 (22.83)	TP53 Wild-Type (%) N = 267 (77.17)
*Age* (years)			
Median (range)	67 (37–87)	67.4 (41–86)	66.9 (37–87)
*Gender, n* (%)			
Female	109 (31.5)	22 (27.8)	87 (32.7)
Male	237 (68.5)	57 (72.2)	180 (67.3)
*Histology, n* (%)			
Adenocarcinoma	233 (67.3)	52 (65.8)	183 (68.3)
Squamous cell carcinoma	96 (27.7)	25 (31.6)	71 (26.4)
Adenosquamous	8 (2.3)	0	8 (2.9)
* Other	9 (2.6)	2 (2.6)	7 (2.6)
*Smoking status*, *n* (%)			
Never	45 (13.0)	5 (6.3)	40 (15)
Current	174 (50.4)	39 (49.3)	135 (50.6)
Past	124 (35.9)	34 (43)	90 (33.8)
Unknown	2 (0.6)	1 (1.4)	1 (0.4)
*ECOG*, *n* (%)			
0	72 (20.9)	19 (24)	53 (19.8)
1	200 (58.0)	41 (52)	159 (59.6)
2+	73 (21.2)	19 (24)	55 (20.6)
*Type of Immunotherapy*, *n* (%)			
Pembrolizumab	205 (59.2)	52 (65.8)	153 (57.4)
Ipilimumab plus nivolumab	141 (40.8)	27 (34.2)	114 (42.6)
*PD-L1 expression*, *n* (%)			
<1%	127 (36.7)	27 (34.1)	100 (37.5)
1–49%	90 (26.0)	15 (19)	92 (34.5)
>50%	129 (37.3)	37 (46.9)	75 (28)

Abbreviation: ECOG, Eastern Cooperative Oncology Group; PD-L1, programmed cell death-ligand 1; TP53, tumor protein p53. Note: Immunohistochemistry was performed on tissue biopsy samples. The tumor’s PD-L1 expression was measured using Ventana’s XT Benchmark (Roche Diagnostics, Basel, Switzerland) using IHC PharmDx (clone 22C3, Dako), UltraView detection kit (FDA approved, Ventana). * Giant cell carcinoma and pleomorphic carcinoma.

## Data Availability

The data either reside within the article itself or can be obtained from the authors upon making a reasonable request.
